# Long access heroin self-administration significantly alters gut microbiome composition and structure

**DOI:** 10.3389/fpsyt.2024.1369783

**Published:** 2024-02-27

**Authors:** Jonathan M. Greenberg, Andrew D. Winters, Branislava Zagorac, David J. Kracht, Dina M. Francescutti, Nazzareno Cannella, Roberto Ciccocioppo, Leah C. Solberg Woods, James Mackle, Gary T. Hardiman, Brittany N. Kuhn, Peter W. Kalivas, Donald M. Kuhn, Mariana Angoa-Perez

**Affiliations:** ^1^ Department of Psychiatry & Behavioral Neurosciences, Wayne State University School of Medicine, Detroit, MI, United States; ^2^ John D. Dingell Veterans Affairs (VA) Medical Center, Detroit, MI, United States; ^3^ Pharmacology Unit, School of Pharmacy, Center for Neuroscience, University of Camerino, Camerino, Italy; ^4^ Department of Molecular Medicine, School of Medicine, Wake Forest University, Winston-Salem, NC, United States; ^5^ School of Biological Sciences and Institute for Global Food Security, Queen’s University Belfast, Belfast, United Kingdom; ^6^ Department of Neuroscience, Medical University of South Carolina, Charleston, SC, United States; ^7^ Department of Physiology, Wayne State University School of Medicine, Detroit, MI, United States

**Keywords:** heroin, gut microbiome, microbial diversity, acquisition, extinction, reinstatement

## Abstract

**Introduction:**

It is well known that chronic opioid use disorder is associated with alterations in gastrointestinal (GI) function that include constipation, reduced motility, and increased bacterial translocation due to compromised gut barrier function. These signs of disrupted GI function can be associated with alterations in the gut microbiome. However, it is not known if long-access opioid self-administration has effects on the gut microbiome.

**Methods:**

We used 16S rRNA gene sequencing to investigate the gut microbiome in three independent cohorts (N=40 for each) of NIH heterogeneous stock rats before onset of long-access heroin self-administration (i.e., naïve status), at the end of a 15-day period of self-administration, and after post-extinction reinstatement. Measures of microbial α- and β-diversity were evaluated for all phases. High-dimensional class comparisons were carried out with MaAsLin2. PICRUSt2 was used for predicting functional pathways impacted by heroin based on marker gene sequences.

**Results:**

Community α-diversity was not altered by heroin at any of the three phases by comparison to saline-yoked controls. Analyses of β-diversity showed that the heroin and saline-yoked groups clustered significantly apart from each other using the Bray-Curtis (community structure) index. Heroin caused significant alterations at the ASV level at the self-administration and extinction phases. At the phylum level, the relative abundance of Firmicutes was increased at the self-administration phase. Deferribacteres was decreased in heroin whereas Patescibacteria was increased in heroin at the extinction phase. Potential biomarkers for heroin emerged from the MaAsLin2 analysis. Bacterial metabolomic pathways relating to degradation of carboxylic acids, nucleotides, nucleosides, carbohydrates, and glycogen were increased by heroin while pathways relating to biosynthesis of vitamins, propionic acid, fatty acids, and lipids were decreased.

**Discussion:**

These findings support the view that long access heroin self-administration significantly alters the structure of the gut microbiome by comparison to saline-yoked controls. Inferred metabolic pathway alterations suggest the development of a microbial imbalance favoring gut inflammation and energy expenditure. Potential microbial biomarkers and related functional pathways likely invoked by heroin self-administration could be targets for therapeutic intervention.

## Introduction

The use of opioid drugs, which includes prescription pain relievers, heroin, and synthetic opioids such as fentanyl, ranges along a continuum from clinical therapeutics to abuse/addiction to fatal overdose. In 2017, 1.7 million Americans suffered from use disorders related to prescription pain relievers and > 650,000 suffered from heroin use disorder (1). In 2019, nearly 50,000 deaths in the USA were attributed to opioid overdose (2, 3). The total economic burden of opioid use disorder and fatal opioid overdose in 2017 was estimated to be $1.02 trillion (4). Therefore, opioid use disorder and overdose constitutes a significant national public health crisis with attendant social and economic impacts. At present, FDA-approved treatments of opioid dependence are limited to buprenorphine, methadone, and naltrexone. However, the steady increase in heroin-related morbidity and mortality over the past two decades in the USA alone and the chronic nature of opioid use disorder points to repeated cycles of cessation of drug use and relapse (5), and the need for more effective and longer lasting treatments.

Opioid pharmacological actions are mediated primarily by a series of opioid receptors expressed by central and peripheral neurons, as well as by neuroendocrine, immune and ectodermal cells (6). The three main opioid receptors in the CNS- mu, delta and kappa- mediate analgesia and euphoria and are sites of action for buprenorphine (partial mu agonist), methadone (mu agonist), and naltrexone (nonselective opioid antagonist). For the most part, these CNS receptors have been the focus of study for understanding the addictive properties of the opioid drugs. However, it is also well known that long-term opioid use disorder is associated with alterations in gastrointestinal (GI) function to include constipation, reduced motility, and increased bacterial translocation by compromising gut barrier function (7–9). These signs of disrupted GI function suggest the possibility that the gut microbiome has been altered (10).

The bulk of the microbiome resides in the GI tract and is composed of bacteria, viruses, archaea, and fungi. It has been estimated that the human GI system contains > 1000 bacterial species and approximately 4 X 10^13^ microorganisms [roughly the same number of human cells (11)], and the gut microbiome expresses ~100 times as many genes as the host human genome (12, 13). Normal functioning of the gut microbiome is essential to the maintenance of human health. A disruption in gut microbiome composition (i.e., dysbiosis) has been linked to numerous diseases, including cancer, diabetes, obesity, immune dysfunction, and inflammatory bowel disease (14, 15). Emerging research is also showing that gut microbiome dysbiosis can play a role in numerous neurologic [e.g., Parkinson disease, Alzheimer disease (16)] and psychiatric conditions [e.g., autism (17), depression, and anxiety (18)], and in eating disorders (19).

Research on gut microbiome involvement in substance use disorders lags well behind most other health disorders, but an increasing number of publications are documenting drug-induced alterations in it and modulation of opioid effects by the gut microbiome. For instance, morphine causes significant alterations in the gut microbiome of animals (9, 20–23) and humans (24–28). The morphine-induced alteration in the gut microbiome causes impairment in reward and sensory responses (29, 30). The gut microbiome has also been shown to mediate morphine analgesic tolerance (31, 32) and withdrawal (33, 34). Exposure to morphine during pregnancy (35) or in newborn rats (36) induces a dysbiosis that persists from the neonatal period into adulthood. Other opioids, including oxycodone (37–39), fentanyl (40, 41), and heroin (22), have also been shown to cause dysbiosis in the gut microbiome. The interactions of opioid drugs with the gut microbiome have been also described in numerous review articles (10, 42–49).

The studies cited above showing gut microbiome alterations in opioid dependence models used noncontingent (e.g., slow-release morphine pellets or minipumps, injection) drug administration. However, it is not known if long-access opioid self-administration has effects on the gut microbiome. Therefore, to simulate contingent opioid use in humans more closely, outbred rats were trained to self-administer heroin using operant schedules of reinforcement. The effect of heroin self-administration on gut microbiome status was determined by 16S rRNA gene sequencing of fecal samples collected before onset of heroin self-administration, at the completion of long-term self-administration, and after post-extinction reinstatement. The results document that long-access heroin self-administration causes a significant disruption of the gut microbiome.

## Materials and methods

### Subjects

Three independent cohorts (N=40 per cohort, 20 males and 20 females) of heterogeneous stock (NMcwiWFsm : HS) rats were used in these studies. Rats were pair-housed initially and left undisturbed in a climate-controlled colony room with a standard 12-hour light-dark cycle for 3 weeks prior to the start of heroin self-administration testing at the Medical University of South Carolina (MUSC). Rats had ad libitum access to food and water. Rats arrived at MUSC at 5 weeks of age, and their first testing/fecal pellet collection occurred at 8 weeks of age. Testing occurred during the dark cycle, between 18:00 h and 6:00 h. During the course of experimentation 47 rats were excluded from the study due to technical issues regarding data collection or to early deaths. The total number of rats tested was 23 (10 male, 13 female), 27 (15 male, 12 female), and 23 (12 male, 11 female) in the three cohorts, respectively. All experimental procedures using animals were approved by the Institutional Care and Use Committee at MUSC. This research was also conducted in accordance with the National Institutes of Health Guide for the Care and Use of Laboratory Animals and the Assessment and Accreditation of Laboratory Animals Care.

### Heroin self-administration

**Figure 1:**
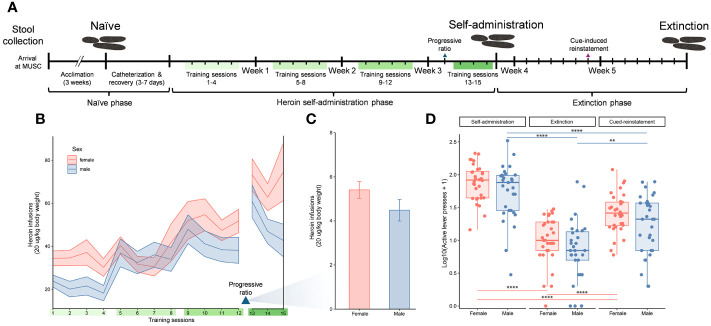
Experimental timeline and indicators of heroin addiction-like behavior. Experimental timeline indicating the three experimental phases of the study (naïve, self-administration, and extinction), self-administration training sessions (green bars), and stool sample collections over the study duration **(A)**. Number of heroin infusions (one infusion = 20 µg/kg of body weight) for male and female rats over 15 training sessions that are indicated with ribbons (mean ± standard error of the mean) **(B)**. Number of heroin infusions by sex during the progressive ratio test (mean ± standard error of the mean) **(C)**. Active lever presses for male and female rats at three distinct timepoints (end of self-administration, extinction and cue induced reinstatement). Data for active lever presses are an average over training sessions 13-15 for the self-administration timepoint, and single day session measurements for the extinction and cued-induced reinstatement timepoints (median ± interquartile range; **p < 0.005, ****p < 0.00005, paired Wilcoxon) **(D)**, similar to the previous panels.

Heroin hydrochloride was obtained from the National Institute on Drug Abuse Drug Supply Program and dissolved in 0.9% sterile saline for use. Indwelling jugular catheters were implanted in each rat under isoflurane anesthesia, with an antibiotic (cefazolin, 0.2 mg/kg, sc) and an analgesic (Ketorolac, 2 mg/kg, sc) administered postoperatively. Animals were given a minimum of 3 days recovery prior to testing (see **Figure 1A**). At the conclusion of experimentation each week, all rats received 0.1 ml of Taurolidine-Citrate Solution (-TCS- Braintree Scientific, Inc) as an antimicrobial and to promote catheter patency. Heroin self-administration was carried out as previously described in detail (49–51) and included saline-yoked controls (4/cohort). Briefly, rats were placed singly into standard operant chambers (Med Associates, St. Albans, VT) outfitted with 2 levers for heroin self-administration. During a session, bar presses on the active lever resulted in presentation of light and tone cues for 5 seconds and an iv infusion of heroin (20 ug/kg/100 ul infusion over 3 seconds). At the time of infusion, the house light turned off for 20 seconds signaling a time-out period whereupon additional presses on the active lever were recorded but without consequence. A session lasted for 12 hours, or terminated once 300 infusions was reached, and a fixed ratio 1 schedule of reinforcement was used. Presses on the inactive lever were recorded but were without consequence. The self-administration phase lasted for 15 sessions (**Figure 1A**). Immediately after the self-administration phase, rats were placed under extinction conditions whereupon presses on either active or inactive levers were recorded but not reinforced with heroin infusions or presentation of the tone/cue light stimuli. A total of 6 extinction training sessions (2 hour/session) occurred. At the conclusion of the extinction phase, rats were exposed to a 2-hour cue-induced reinstatement test whereby active lever presses resulted in tone/cue-light presentation and pump activation but no heroin infusion. Fecal boli were collected from the operant chamber of each subject before the start of self-administration, after completion of the heroin long-access self-administration phase (after training session 15) and after extinction and cued reinstatement phases (**Figure 1A**). Fecal boli were collected immediately after the indicated sessions using sterile forceps, placed into sterile plastic tubes, and frozen immediately at -80°C. Thereafter, fecal boli were shipped on dry-ice to Wayne State University for 16S rRNA gene sequencing and analysis. Analyses of the number of heroin infusions over the self-administration period were carried out using a mixed-effects model (REML) with sex, heroin training sessions and the interaction between sex and training session as variables. Comparisons at the progressive ratio test (average number of infusions) were performed with student’s t tests. The log-normalized number of active-lever presses at the end of heroin self-administration, after extinction and at cue-induced reinstatement in males and females were compared using the Friedman one-way repeated measure ANOVA followed by Wilcoxon *post hoc* comparisons.

### Gut microbiome analysis

Fecal samples were collected and processed for sequencing of the 16S rRNA gene. These samples, along with the appropriate positive/negative controls and mock community, were processed for amplicon metagenomic library preparation and sequencing using an Illumina MiSeq system as we have previously described (50–54). The V4 region of the 16S rRNA gene was amplified and sequenced via the dual indexing strategy developed by Kozich et al. (55). 16S rRNA gene sequences were clustered into amplicon sequence variants (ASVs) using the Divisive Amplicon Denoising Algorithm (DADA2) (56) pipeline to obtain merged, denoised, chimera-free, inferred ASVs, with the exception that forward and reverse reads were truncated at 240 and 150 bases, respectively. Sequences were then classified using the silva_nr_v132_train_set database with a minimum bootstrap value of 80%, and sequences that were derived from Archaea, chloroplast, Eukaryota, mitochondria, or that could not be classified to a bacterial phylum were removed. All samples were normalized to 11,377 reads using the rarefy function in phyloseq R package (v 1.44.0). Good’s coverage was ≥ 97.9% for all samples and contained a total of 2314 ASVs from 14 bacterial phyla. For analyses, 219 bacterial profiles were used from 73 rats with samples from each experimental phase (naïve, self-administration, extinction). α-diversity of bacterial profiles was characterized using Chao1, Shannon, and Inverse Simpson indices calculated via the phyloseq package. Differences in α-diversity were statistically evaluated through linear modeling using base R (v 4.3.1) and three-way ANOVAs factoring cohort, treatment, and sex, using the R package car (v 3.1.2). β-diversity was characterized using the Bray-Curtis dissimilarity index based on relative abundance data (i.e., percent abundance), calculated via the vegan R package (v 2.6.4). Three-dimensional principal coordinate analysis (PCoA) plots were used to visualize the similarity of the sample profiles with the car package (v 3.1.2). To account for any potential effect of individual rat identity, cohort variation, or sex on the gut microbiome, these variables were controlled for within and across experimental phases, as appropriate. Changes in β-diversity were statistically evaluated with non-parametric MANOVAs (NPMANOVAs) using the vegan package. R^2^ values were also included to indicate the percentage of variation in the response that was explained by each model utilized. Differential relative abundance of bacterial ASVs and phyla between the heroin and saline-yoked controls and between sexes were assessed using a negative binomial model as implemented in the R package Microbiome Multivariable Associations with Linear Models (MaAsLin2, v 1.14.1). MaAsLin2 is an established methodology to assess multivariable association of microbial community features with complex metadata in population-scale observational studies (57). A minimum prevalence of 0.40 was used and multiple comparisons were adjusted using the Benjamini-Hochberg method. Values for q < 0.05 were deemed statistically significant.

### Inference of functional genes and pathways

PICRUSt2 software package version 2.5.2 (58) was used for predicting functional pathway occurrence based on marker gene sequences (16S rRNA sequencing data). MetaCyc ontology predictions (59) were used for metabolic pathway classification and were compared by Statistical Analysis of Metagenomic Profiles (STAMP) (60) using the Welsh’s t-test (filtered p > 0.05) corrected for multiple tests with Benjamini-Hochberg. Functional pathways were then annotated based on the individual MetaCyc Superpathways.

## Results

### Heroin intake at self-administration and addictive-like behaviors

Analysis of the number of heroin infusions over the self-administration period showed a significant escalation in drug intake across the 12 training sessions (F_4.328, 250.4_ = 15.18, p < 0.05). The ranges of heroin intake were 0-5.2 mg/kg in females and 0-3.74 mg/kg in males. This drug escalation was independent of sex as both males and females showed a similar pattern of increase (**Figure 1B**). The interaction between sex and training sessions was not significant. Following the training sessions, a progressive ratio test was performed to determine the motivation for the rats to work for the drug. In this test the effort needed to retrieve an infusion reward increased across trials. While comparisons between the males and females in the number of infusions at this test showed a reduced response in males, this was not statistically significant (**Figure 1C**). Addiction-like behaviors in males and females were also evaluated by comparing the number of active lever presses at the end of self-administration, extinction and cue induced reinstatement (F_r_ = 44.1, df 2, p < 0.0001 for males; F_r_ = 54, df 2, p < 0.0001 for females) (**Figure 1D**). The number of active lever presses decreased significantly at the extinction phase compared to the end of self-administration training (p < 0.00001 for both sexes), but increased at cue-induced reinstatement (p < 0.00001 for females, p = 0.001 for males). Similar to the escalation of heroin intake, behaviors indicating addiction-like patterns were shown in both sexes but no statistical differences were detected by sex.

### Alterations in gut microbiome diversity by heroin self-administration

**Figure 2:**
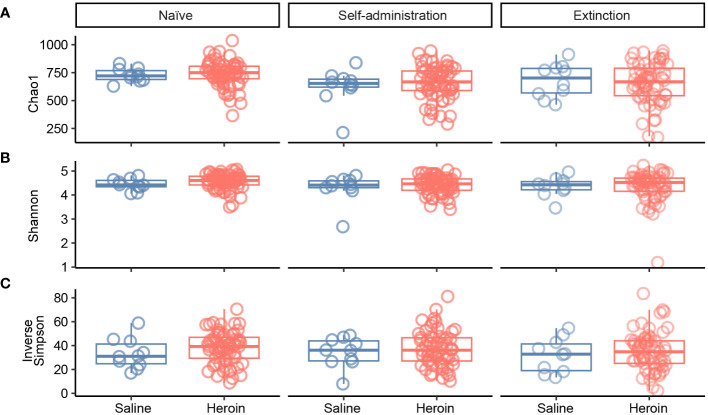
Effects of heroin self-administration on α-diversity. The α-diversity metrics Chao-1 **(A)**, Shannon **(B)** and Inverse Simpson **(C)** were determined for 16S rRNA gene sequencing profiles of rat fecal samples from each of the experimental phases. Data are presented as median ± interquartile range (line through box, respectively) and include data from each subject. No statistical significances were detected.

**Figure 3:**
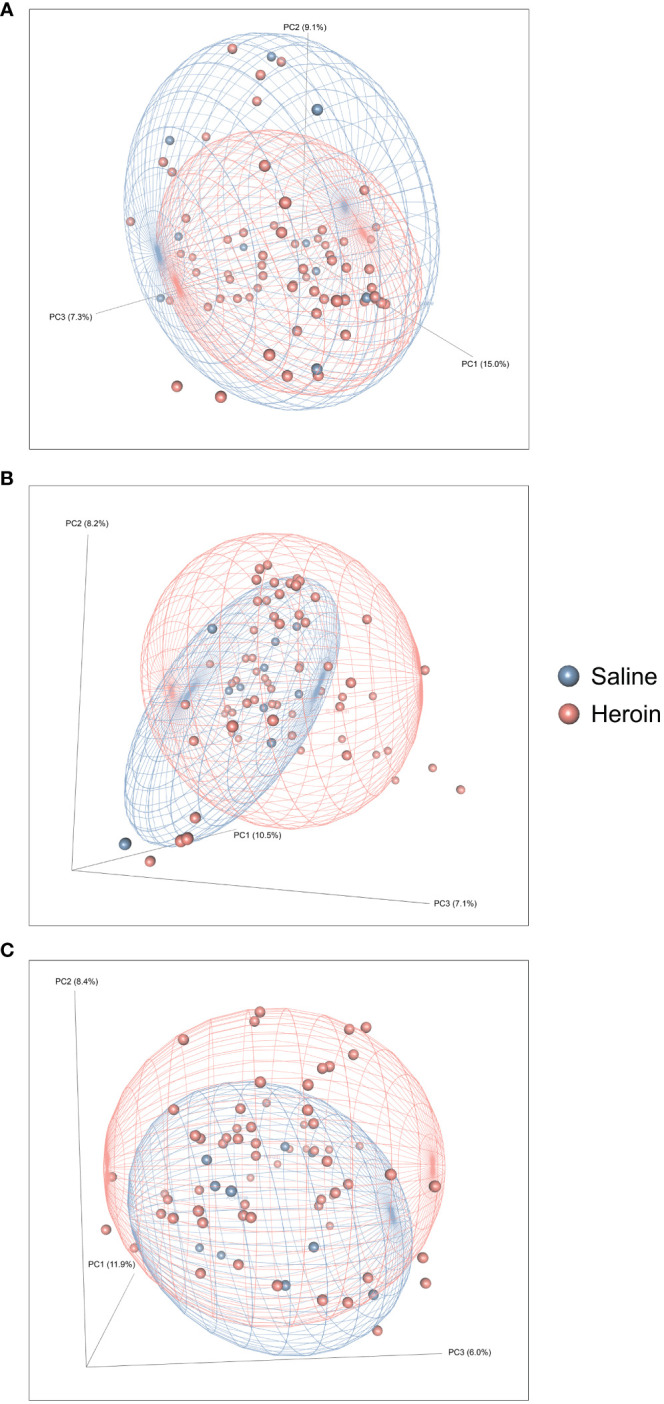
Effects of heroin self-administration on β-diversity. Principal Coordinates Analysis (PCoA) plots of the bacterial profiles from the 16S rRNA gene sequencing at the naïve **(A)**, self-administration **(B)**, and extinction **(C)** phases. Percentages along the X and Y axes indicate the amount of variance explained for principal coordinates 1 through 3, respectively. PCoAs were generated using the Bray-Curtis index. Ellipses indicate 80% confidence intervals and individual data points are color-coded by group (blue = saline-yoked controls; red = heroin self-administration). The plot orientations were chosen for clarity of the data in three-dimensional space.

**Figure 4:**
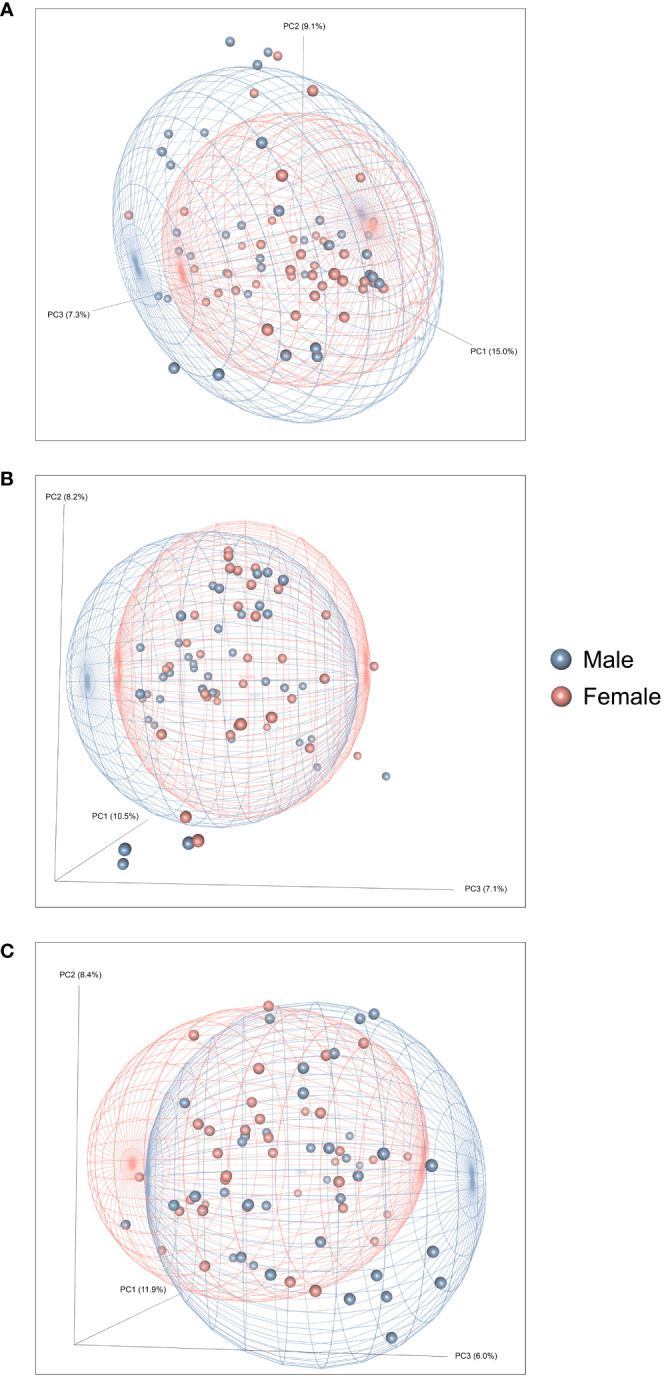
Effects of heroin self-administration on the β-diversity of the gut microbiome in males and females. Principal Coordinates Analysis (PCoA) plots of the bacterial profiles from the 16S rRNA gene sequencing at the naïve **(A)**, self-administration **(B)**, and extinction **(C)** phases. Plots represent the same data as in **Figure 2**, but are color-coded by sex (red circles = females; blue boxes = males). Percentages along the X and Y axes indicate the amount of variance explained for principal coordinates 1 through 3, respectively. PCoAs were generated using the Bray-Curtis index. Ellipses indicate 80% confidence intervals and are color-coded by sex. The plot orientations were chosen for clarity of the data in three-dimensional space.

**Figure 2** presents the analyses of the effects of long-access self-administration on microbial α-diversity. Three-way ANOVA analyses revealed that heroin did not change α-diversity in any of the experimental phases as assessed using the Chao-1 (richness, panel A), Shannon (evenness, panel B), and Inverse Simpson (heterogeneity, panel C) indices. Results from analyses of β-diversity of the bacterial profiles are presented in **Figure 3**. An initial NPMANOVA analysis controlling for experimental phase showed significant main effects for heroin self-administration (F_1,146_ = 2.155, p = 0.001, R^2 ^= 0.009), sex (F_1,146_ = 2.04, p = 0.0009, R^2 ^= 0.011) and rat identity (F_70,146 _= 1.375, p = 0.0009, R^2 ^= 0.401). No significant interactions were detected between any of these factors. Given that the overall effects of heroin self-administration were statistically significant, the effects of each experimental phase were subsequently tested with NPMANOVAs while controlling for cohort. Heroin self-administration, sex, or self-administration X sex interactions were not found at the naïve phase (i.e., before initiation of heroin self-administration) (**Figure 3A**). At the self-administration phase, heroin self-administration (F_1,69 _= 1.45, p = 0.03, R^2 ^= 0.02) and sex (F_1,69 _= 1.57, p = 0.01, R^2 ^= 0.02) were both significant but the interaction between self-administration and sex was not (**Figure 3B**). At the extinction phase, heroin self-administration (F_1,69 _= 1.61, p = 0.007, R^2 ^= 0.02), sex (F_1,69 _= 1.7, p = 0.005, R^2 ^= 0.02) and the interaction between self-administration and sex (F_1,69 _= 1.65, p = 0.002, R^2 ^= 0.02) were significant (**Figure 3C**). For clarity purposes, PCoAs displaying sex effects for each experimental phase are presented in **Figure 4A** (Naïve), 4B (heroin self-administration), and 4C (extinction).

### MaAsLin2 analysis for biomarkers

**Figure 5:**
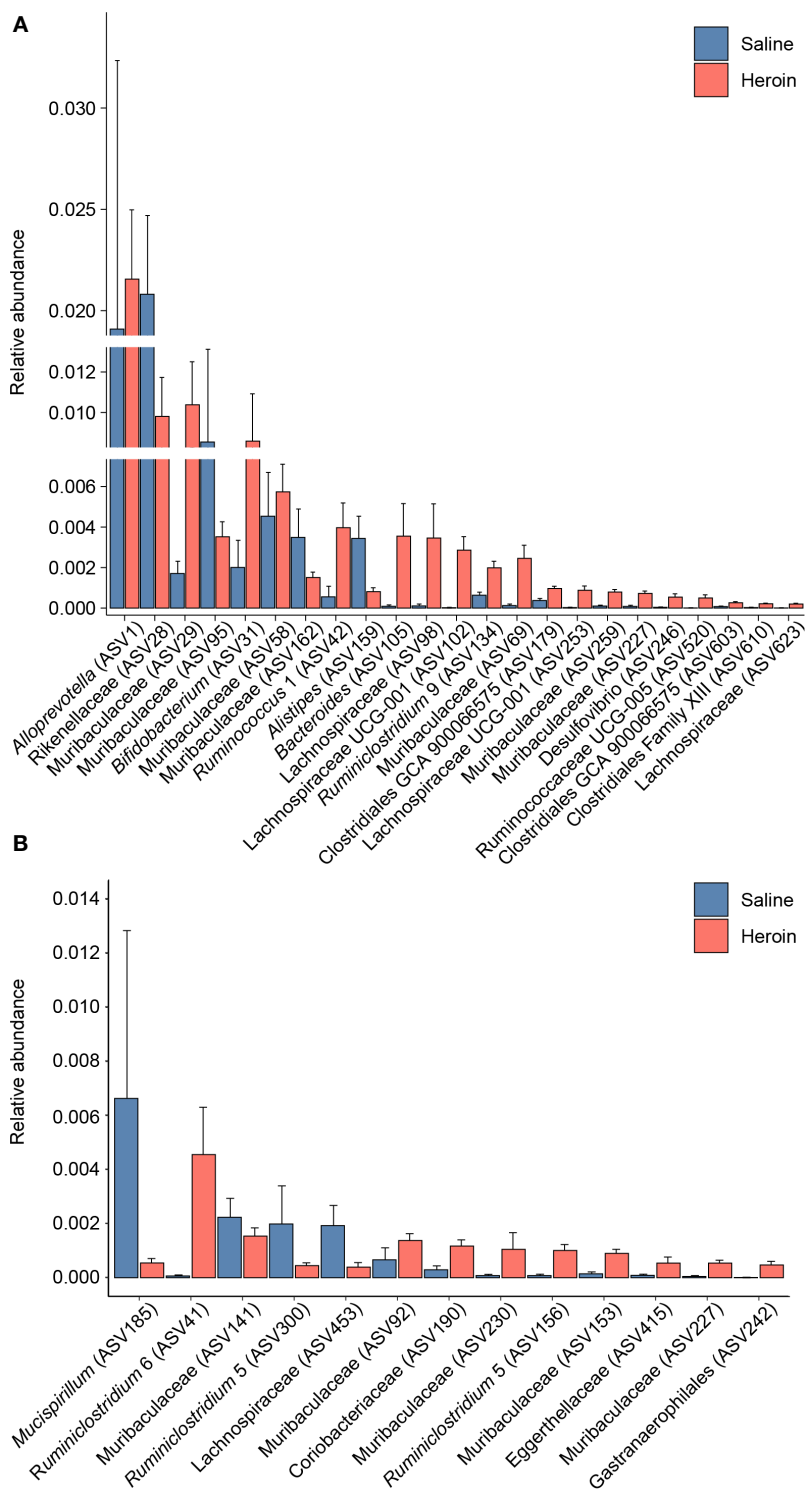
Bacterial taxa differentially abundant after heroin self-administration. Relative abundance of taxa identified as increased or decreased after the heroin self-administration phase when compared to saline-yoked controls **(A)**. Relative abundance of taxa differentially abundant following the extinction phase **(B)**. Bars indicate mean relative abundance ± SEM. All taxa depicted are statistically significant (q < 0.05) as assessed via MaAsLin2.

**Figure 6:**
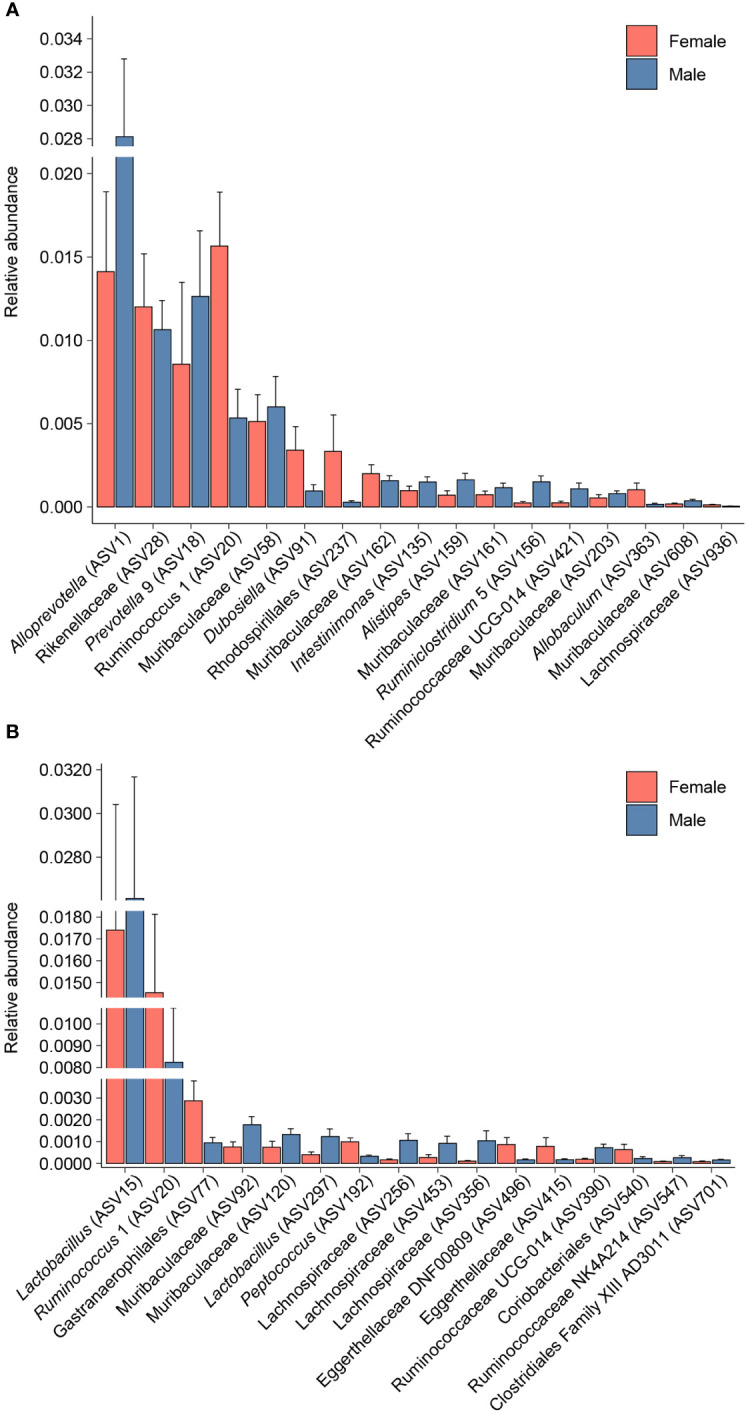
Bacterial taxa differentially abundant after heroin self-administration by sex. Relative abundance of taxa identified as increased or decreased in males and females after the heroin self-administration phase **(A)**. Relative abundance of taxa differentially abundant in males and females following the extinction phase **(B)**. Bars indicate mean relative abundance ± SEM. All taxa depicted are statistically significant (q < 0.05) as assessed via MaAsLin2.

Differential abundance analysis with MaAsLin2 at the ASV level identified 23 bacterial ASVs that were significantly altered at the self-administration phase (**Figure 5**). Among these, 19 bacterial ASVs were increased by heroin self-administration and 4 ASVs were decreased compared to saline-yoked controls (**Figure 5A**). Of the 23 ASVs that emerged from this analysis, most could be identified at the taxonomic level of family (N=13) with fewer identified at the level of genus (N=7) and order (N=3). In addition, the majority of these taxa (91%) are in the phyla of Bacteroidetes (N=11) and Firmicutes (N=10). Those taxa that were increased to the greatest extent by heroin self-administration included Bacteroides (ASV105), Lachnospiraceae (ASV102), and Muribaculaceae (ASV69) while those associated with greater decreases after heroin self-administration included Rikenellaceae (ASV28), Muribaculaceae (ASV95) and Alistipes (ASV159). At the extinction phase, 13 ASVs were found significantly altered by heroin self-administration. Among these, 9 ASVs were increased and 4 were decreased in the heroin group (**Figure 5B**). Of these ASVs, most were identified at the taxonomic levels of genus (N=6) and family (N=6). The majority of these ASVs associated with the extinction phase are in the phyla of Bacteroidetes (N=5) and Firmicutes (N=4). Those taxa increased to the greatest extent in the heroin self-administration group included *Ruminoclostridium 6* (ASV41), Muribaculaceae (ASV230) and *Ruminiclostridium 5* (ASV156) while those showing the greatest decreases included *Mucisprillum* (ASV185), *Ruminiclostridium 5* (ASV300) and Lachnospiraceae (ASV453). MaAsLin2 also revealed 17 bacterial ASVs differentially abundant by sex at the self-administration phase (**Figure 6A**). Of the 17 ASVs, ten were increased in males, while seven were increased in females. Of the 17 ASVs that emerged from this analysis, most could be identified at the taxonomic level of genus (N=8) with fewer identified at the level of family (N=7) and order (N=2). Similarly, in the extinction phase, sixteen bacterial ASVs were differentially abundant, with 10 higher in males, and 6 higher in females (**Figure 6B**). Notably, only one ASV was detected as differentially abundant in both phases and was higher in females, *Ruminococcus 1* (ASV20). Similarly to the treatment comparisons, the majority of taxa (85%) differentially abundant between the sexes were of the phyla Bacteroidetes (N=11) and Firmicutes (N=17).

### Analysis of the gut microbiome in heroin self-administration at the phylum level

**Figure 7:**
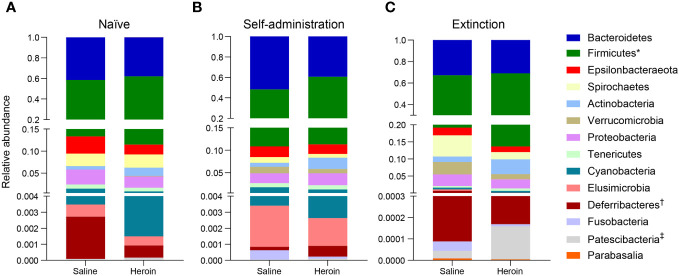
Effects of different phases of heroin self-administration on bacterial phyla. Relative abundance of the heroin and saline-yoked groups at the naïve **(A)**, self-administration **(B)**, and extinction **(C)** phases. Data are presented as mean relative abundance. The symbols indicate: *q < 0.05 for Firmicutes at the self-administration phase; ^†^ q< 0.005 for Deferribacteres at the extinction phase; and ^‡^q < 0.05 for Patescibacteria at the extinction phase.

**Figure 8:**
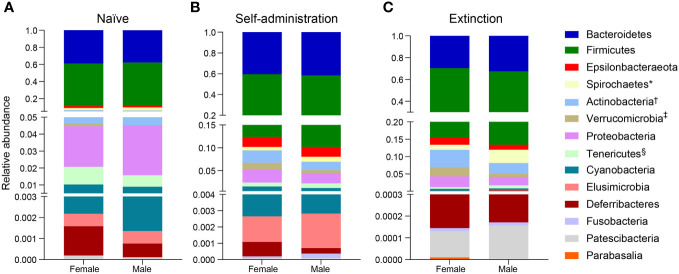
Effects of different phases of heroin self-administration on bacterial phyla in males and females. Relative abundance of bacterial phyla in males and females at the naïve **(A)**, self-administration **(B)**, and extinction **(C)** phases. Data are presented as mean relative abundance. The symbols indicate: *q < 0.05 for Spirochaetes at the extinction phase; ^†^q < 0.005 for Actinobacteria at the self-administration and extinction phases; ^‡^q < 0.005 for Verrucomicrobia at the naïve phase, and ^§^q < 0.05 for Tenericutes at the extinction phase.

Broad assessment of the bacterial communities at the phylum level revealed that Bacteroidetes and Firmicutes accounted for >90% average relative abundance (**Figure 7**), regardless of experimental group (saline, heroin) or experimental phase (naïve, self-administration, extinction). MaAsLin2 was used to identify specific phyla that were differentially abundant between the heroin and saline groups. No phyla were differentially abundant between groups at the naïve phase (**Figure 7A**). Firmicutes was the only differentially abundant phylum at the self-administration phase (q < 0.05; **Figure 7B**) and was higher in the heroin group when compared to the saline-yoked controls (49.3% vs 37.4%). At the extinction phase (**Figure 7C**), two phyla were shown to be significantly altered by heroin (q < 0.05). In the heroin group, Deferribacteres was less relatively abundant (0.05% vs 0.66%) and Patescibacteria was more relatively abundant compared to saline-yoked controls (0.015% vs 0.004%). Similarly, to above, Bacteroidetes and Firmicutes accounted for >84% average relative abundance for each sex, regardless of phase, however several lower abundance phyla were differentially abundant by sex (**Figure 8**). Prior to heroin self-administration, Verrucomicrobia was higher in females (0.16% vs 0.02%) and persisted following heroin self-administration (1.44% vs 0.63%) increasing in average relative abundance across each phase for both sexes (**Figures 8A, B**). After extinction, the sex difference was no longer significant (2.7% vs 0.94%). Actinobacteria was also higher in females at the self-administration phase (2.7% vs 1.8%) as well as the extinction phase (5.0% vs 3.0%). In the extinction phase, Spirochaetes and Tenericutes were higher in males (Spirochaetes: 3.9% vs 1.5%; Tenericutes: 0.91% vs 0.51%) (**Figure 8C**).

### Predictive functional profiling of heroin self-administration administration

**Figure 9:**
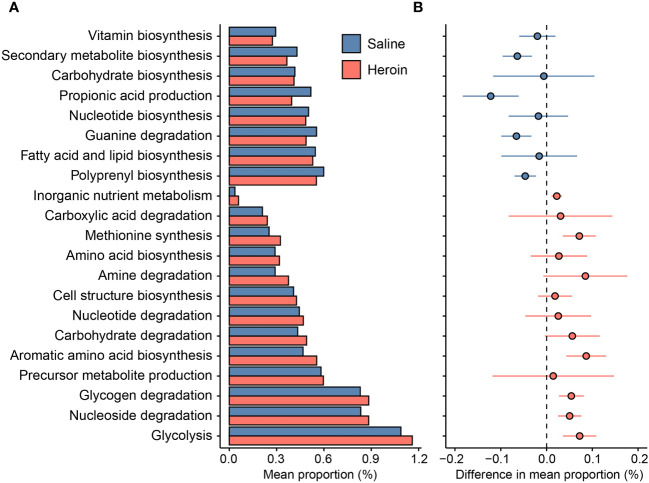
Differences in the predicted functions of the metagenome from 16S rRNA gene sequencing of fecal samples from rats exposed to heroin. Functional pathways were annotated based on the individual MetaCyc Superpathways, showing the mean proportion (%) of the pathways identified **(A)** and the magnitude of difference in the pathway proportion between heroin and saline-yoked **(B)**. Horizontal lines through individual data points in **(B)** indicate standard error of the mean. Negative values indicate pathways less abundant in heroin relative to saline-yoked (blue); Positive values indicate pathways more abundant in heroin (red).

The results from 16S rRNA gene sequencing were analyzed by PICRUSt2 (61) to predict the functional composition of the current database as shown in **Figure 9**. A total of 176 of the 354 (49.7%) functional pathways predicted to be present in the saline-yoked and heroin fecal metagenomes differed significantly between the two treatment groups. After annotating the functional pathways based on the individual MetaCyc Superpathways, it was revealed that 8 of the 21 (38.1%) predicted functions were decreased in heroin compared to saline-yoked controls. These pathways included the biosynthesis of vitamins, energy secondary metabolites, carbohydrates, propionic acid and nucleotides, and the degradation of guanine. Complementarily, 13 of the 21 (61.9%) functional groups were increased in heroin samples compared to saline-yoked controls. These pathways included the degradation of carboxylic acids, amines, nucleotides, carbohydrates, glycogen and nucleosides, and the biosynthesis of methionine, amino acids, cell structure, and aromatic amino acids, as well as the metabolism of inorganic nutrients, precursor metabolite production, and glycolysis. PICRUSt2 analyses comparing males and females revealed that heroin administration was not associated with sex-dependent changes in functional profiling.

## Discussion

The goal of the present study was to determine if long access heroin self-administration disrupts the gut microbiome in outbred heterogeneous rats. The NMcwiWFsm : HS rats are outbred rodent populations that are known for their high genotypic and phenotypic variability. This strain closely resembles the variation found in human populations (62), and increases the translational relevance of this study. The escalation of heroin intake across the self-administration training sessions, the decrease in active lever responses at extinction, and the recovery of these responses at cue-induced reinstatement, indicate the presence of addiction-like behaviors, rather than non-disordered opioid use. However, these responses were similar in males and females, indicating that both sexes are responsive to the rewarding and reinforcing effects of heroin self-administration. This was consistent with a lack of sex differences in opioid intake at fixed and progressive ratios reported by Ren and Lotfipour in a fentanyl self-administration model (63). While sex effects were not observed in heroin intake, our results show that the gut microbiome was significantly altered after the self-administration and extinction/reinstatement phases of contingent heroin intake by comparison to saline-yoked controls. Heroin did not alter α-diversity of the gut microbiome at any of the experimental phases but it did significantly alter β-diversity in the self-administration and extinction/reinstatement phases. Sex differences were also observed for β-diversity during the self-administration and extinction/reinstatement phases. These differences were not detected prior to heroin use, which is consistent with previous reports that the gut microbiome does not vary by sex in control rats (64, 65). These changes in β-diversity confirm that the structure of the gut microbial community was changed significantly by heroin self-administration. Furthermore, these differences in the gut microbiome structure across the experimental phases varied by sex (**Figures 4B, C**). These results contrast with the study by Ren and Lotfipour, where sex differences were found after opioid self-administration in α-diversity but not in β-diversity. Given that baseline sex variances in α-diversity were reported in Sprague Dawley rats by these authors, it is possible that these differences could be explained by the divergence in drug and rat strains.

To expand the examination of the consequences of drug-induced alterations in β-diversity, the effects of heroin self-administration on the taxonomic makeup of the gut microbiome were examined. Changes at the ASV (**Figure 5**) and phylum levels (**Figure 7**) of analysis for the self-administration phase were in close agreement and showed that Bacteroidetes (48%) and Firmicutes (43%) were the taxonomic levels at which the majority (>90% together) of alterations were observed. This pattern was expected in light of the fact that the rat microbiome is dominated by these two phyla (66). The relative abundances of the genus *Bacteroides* as well as the Lachnospiraceae and Muribaculaceae families were increased to the greatest extent during the self-administration phase. Members of the Bacteroides genus have numerous commensal effects, yet many are pathogenic. *B. fragilis* is very commonly found in infections and after disruption of the intestinal wall or other perforations (67). This anaerobic species often participates in secondary phases of infection of the gut following the acute phase of infection by *E. coli* (67). These pathogenic roles of the Bacteroides genus are consistent with the opioid-induced potentiation of infection virulence and increases in systemic bacterial dissemination (68), exacerbation of Gram-positive sepsis (69), as well as the impairment of intestinal epithelial repair in HIV-infected humanized mice (70). Members of the Lachnospiriceae family, such as those of the *Bacteroides* genus, have beneficial effects including production of short chain fatty acids and maturation of the immune system. However, this family has also been implicated in several disease states, most of which are characterized as inflammatory conditions (e.g., metabolic disease, irritable bowel disease (71)). This is consistent with the known ability of morphine to cause gut barrier disruption and systemic inflammation (9). The alterations in Muribaculaceae in the heroin self-administration phase is paradoxical. A total of 7 different Muribaculaceae ASVs were significantly altered in this phase and 5 of these were increased in relative abundance while 2 were decreased. Muribaculaceae abundance is negatively correlated with inflammation (72) and its abundance has been shown to decline in morphine treated humanized HIV rats and their controls (73). This present result is difficult to link to heroin specifically as increases in the Muribaculaceae would be expected to counteract opioid induced gut inflammation. On the other hand, it is possible that these increases represent a protective response that was of insufficient magnitude to alter heroin self-administration. The differences in the taxonomic makeup of the gut microbiome across the heroin phases by sex were also examined at the ASV (**Figure 6**) and phylum levels (**Figure 8**). While most of the bacterial taxa differentially abundant in heroin vs saline-yoked controls at the ASV level were also identified by sex, some ASVs were exclusive to sex. This is the case for *Prevotella 9* (ASV18) and *Dubosiella* (ASV91) for the self-administration phase, as well as *Lactobacillus* (ASV15, ASV297) and Gastranaerophiliales (ASV77) for the extinction phase (**Figure 6**). Likewise, comparisons in phyla structure in males and females across phases identified taxa that were specifically changed by sex, but not by heroin treatment. Members of Spirochaetes, Actinobacteria, Verrucomicrobia and Tenericutes were differentially abundant in males compared to females (**Figure 8**). These results contribute to the sex-dependent differences in β-diversity identified in each experimental phase.

At the end of the extinction/reinstatement phase, fewer taxa were altered in relative abundance (N=13) than in the self-administration phase (N=23), possibly reflecting a rebalancing of the gut microbiome after cessation of heroin intake. The Muribaculaceae family showed the greatest number of changes in the extinction/reinstatement phase, with 4 showing increased abundance and one showing a decrease. The taxa showing the greatest increases in extinction were *Ruminiclostridium 6* and Coriobacteriaceae. *Ruminoclostridium 6* has been associated with high fat diet (74) and diabetes-induced inflammation (75), and Coriobacteriaceae abundance is increased significantly in irritable bowel disease (76). This finding is consistent with opioid-induced disturbances in gut inflammation (9, 31) and suggests that this effect is quite persistent. Significant decreases in *Mucispirillum* were observed in the extinction/reinstatement phase as well. *Muscispirillum schaedleri* has been shown to protect the gut from colitis (77) suggesting that the large reduction in its abundance in the extinction/reinstatement phase could contribute further to gut inflammation. Similarly, the decrease in *Ruminiclostridium 5* would be predicted to worsen gut health because this genus is a short chain fatty acid producer (78).

PICRUSt2 analysis was undertaken to predict metabolic alterations that would be expected based on bacterial marker gene sequences. While the low variance of microbial functional profiles can affect the accuracy of PICRUSt2 base metrics, and degrade its performance outside of human datasets (79), there are advantages in using this tool. PICRUSt2 is becoming increasingly popular in microbial ecology studies as it contains an updated and larger database of gene families and reference genomes than other similar tools (58, 80). This predictive tool allows for initial exploration of hypotheses prior to shotgun sequencing or metabolomics analyses. In this study, PICRUSt2 was used in an exploratory manner and confirmatory studies can follow. The results from this analysis were in consensus with the literature on changes in opioid-induced metabolomics measured from metabolites in serum and urine [see review by Dinis-Oliveira (81, 82)]. Pathways predicted to be reduced by the PICRUSt2 analysis have been documented in studies on opioid-induced metabolomics to include vitamin biosynthesis (83, 84), carbohydrate biosynthesis (85, 86), propionic acid production (87), nucleotide biosynthesis (81, 88), guanine degradation (89) and fatty acid and lipid biosynthesis (84, 85). Those pathways predicted to increase also aligned with existing literature on opioid induced metabolomics alterations. These include amino acid and amine biosynthesis (85, 86, 89–91), nucleotide and nucleoside degradation, and increased depletion of energy sources (82, 89). Indicators of oxidative stress related to opioid-induced increases in lipid peroxidation and membrane disruption (89) were also consistent with the PICRUSt2 predictions. Together, pathways predicted by PICRUSt2 to be increased or decreased by heroin and other opioids suggest an overall reduction in the tricarboxylic acid cycle, increased energy expenditure and altered amino acid metabolism, and agree with alterations in opioid metabolomic seen *in vivo* (81, 89). These results add a new dimension to existing metabolomics research on opioids by suggesting at least some of these changes are mediated at the level of the gut microbiome.

The magnitude of the gut microbiome changes resulting from long-access heroin self-administration seen presently appeared to be smaller than observed in other studies of opioid-induced dysbiosis, particularly as it relates to the relative abundance of taxa. The total dose of heroin self-administered in the present experiment amounted to approximately 10 mg/kg over 15 days, or an average daily dose of 0.75 mg/kg/day. Despite the lower doses achieved with heroin self-administration, this method allowed for the study of volitional use of heroin. Other studies used higher doses (20-80 mg/kg) and administered opioids via slow-release pellets or minipumps, or by systemic injection (20–23). These results agree with the observation by Taylor and colleagues (29) showing that differing opioid regimens influence the gut microbiome in a specific manner. The results of our study also agree with previous studies showing significant alterations in β-diversity (20, 22, 31) without changes in α-diversity (21, 22) after opioid administration.

This study has a number of strengths. First, it is the first to study to assess the effects of heroin self-administration on the gut microbiome. Second, the use of a long-access, contingent heroin self-administration model with heterogeneous stock rats as subjects to better reflect behavioral and genetic variance associated with human opioid abuse. Third, it recapitulates the sex dependent effects of opioids on various pharmacological and behavioral outcomes. Fourth, it establishes similarity of the effects of heroin with those of morphine and other opioids on the gut microbiome and potentially identifies biomarkers for heroin self-administration using MaAsLin2 analysis. Weaknesses of this study include sequencing at the 16S rRNA level which does not always allow identification of altered bacteria at the species level (as opposed to whole genome sequencing), lack of analyses of gut inflammation and function, and lack of a correlation between gut microbiome changes and behavioral outcomes such as heroin intake. Additional studies are underway to address these weaknesses.

## Data availability statement

The raw data supporting the conclusions of this article was deposited in the SRA database, BioProject # PRJNA1066745.

## Ethics statement

The animal study was approved by Institutional Care and Use Committee at Medical University of South Carolina. The study was conducted in accordance with the local legislation and institutional requirements.

## Author contributions

JG: Data curation, Formal analysis, Writing – original draft, Writing – review & editing. AW: Writing – review & editing. BZ: Data curation, Writing – review & editing. DJK: Writing – review & editing. DF: Writing – review & editing. NC: Writing – review & editing, Methodology. RC: Methodology, Writing – review & editing. LS: Writing – review & editing. JM: Writing – review & editing, Data curation. GH: Writing – review & editing, Investigation. BK: Writing – review & editing, Formal analysis, Investigation, Methodology, Resources. PK: Formal analysis, Investigation, Methodology, Writing – review & editing, Resources. DMK: Formal analysis, Investigation, Methodology, Writing – review & editing, Conceptualization, Funding acquisition, Resources, Writing – original draft. MA-P: Conceptualization, Formal analysis, Methodology, Writing – original draft, Writing – review & editing, Data curation, Investigation, Supervision.
